# Supplementary material for: Saccharomyces cerevisiae Rhodanese RDL2 Uses the Arg Residue of the Active-Site Loop for Thiosulfate Decomposition
Source: Antioxidants (Basel). 2021 Sep 26;10(10):1525. doi: 10.3390/antiox10101525 (PMC8533005; doi:10.3390/antiox10101525)
Supplement: Supplementary file 1 [file antioxidants-10-01525-s001.zip › antioxidants-1369925-supplementary.pdf]

Saccharomyces cerevisiae rhodanese RDL2 uses the Arg residue of the active-site loop for thiosulfate decomposition

Qingda Wang[1], Huanjie Li[2], Yongzhen Xia[1], Luying Xun[1,3], Huaiwei Liu[1*]

[1]State Key Laboratory of Microbial Technology, Shandong University, Qingdao, 266237, PR China;

[2]School of Medicine, Cheeloo College of Medicine, Shandong University, Jinan, 260024, China

[3]Department of Chemistry, School of Molecular Biosciences, Washington State University, Pullman, WA, 99164-4630, USA.

Running title: *The loop-ending amino acids are critical for rhodaneses activities*

*Co-correspondence: liuhuaiwei@sdu.edu.cn; Tel. + 81 532 58631572.

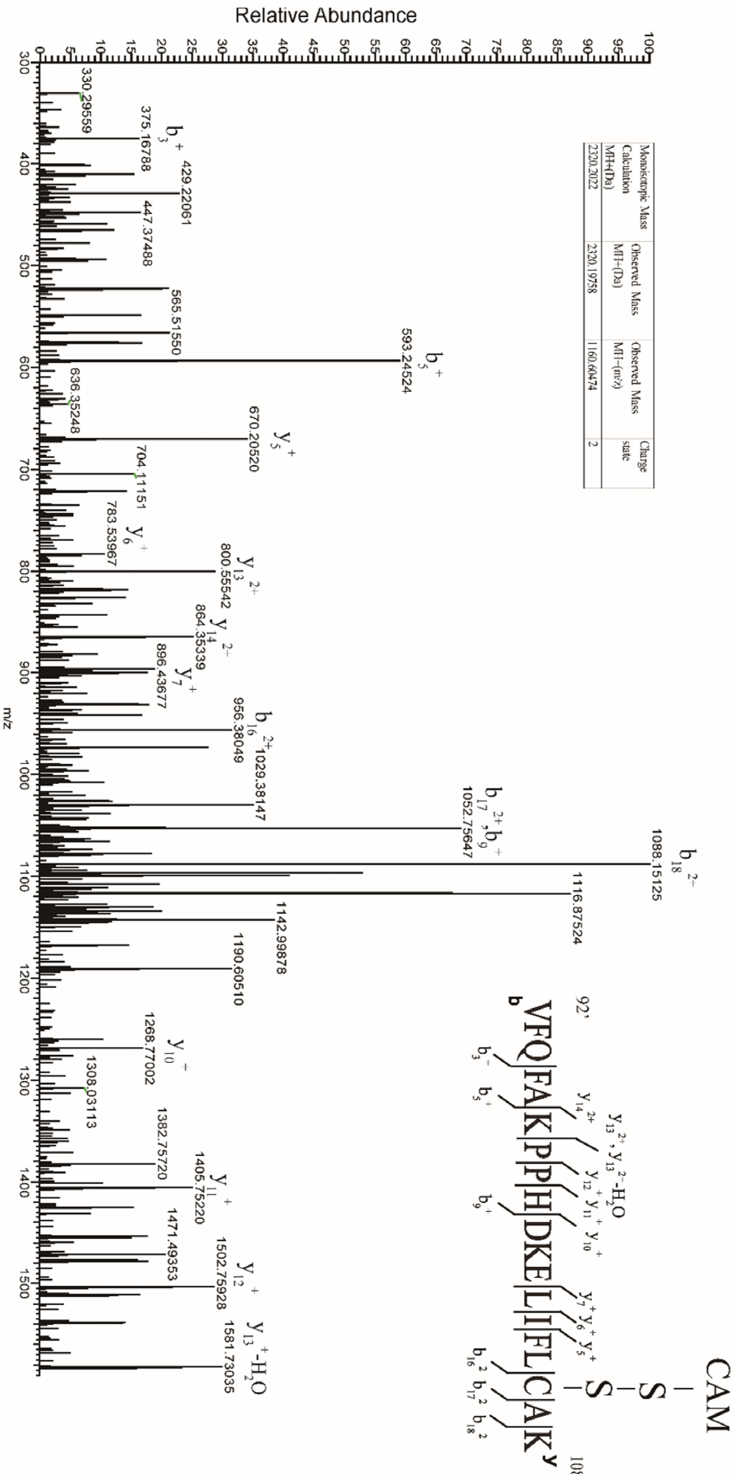

Figure S1. LTQ-Orbitrap tandem mass spectrometry analysis of Cys106 modification in thiosulfate-reacted RDL2.

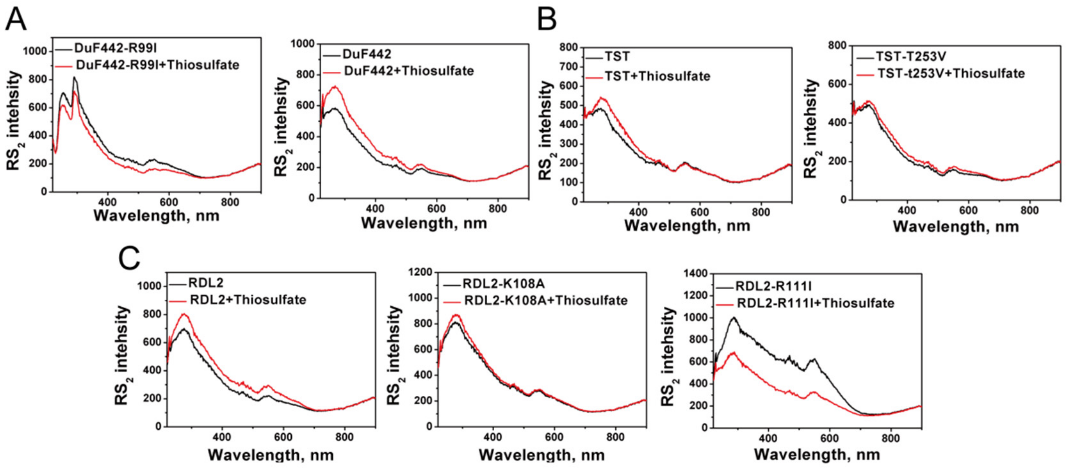

Figure S2. RS_2 analysis of unreacted- and thiosulfate-reacted rhodanese. ΔRS_2 at a specific wavelength was calculated by $\Delta RS_2{}^{w}{}_{\mathrm{reacted}}$ -$\Delta RS_2{}^{w}{}_{\mathrm{unreacted}}$ (*w* is the wavelength). Total ΔRS_2 was calculated by adding up all ΔRS_2 values at 240 nm~550 nm range.

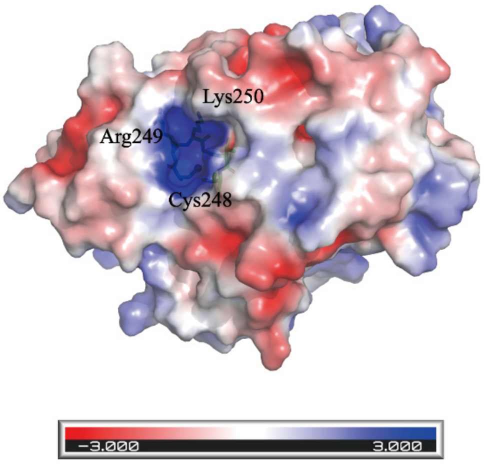

Figure S3. **Modeled 3D structure of TST.** The structure of DUF442 was generated by SWISS-MODEL using the *Bos taurus* rhodanese as a template (PDB entry:1DP2) (90.7% sequence similarity). The 3D homology modeling structure of TST showed that its active

center is located in a pocket with positive electrostatic field. Lys_{250} and Arg_{249} on the active site loop are major contributors to the positive electrostatic field.

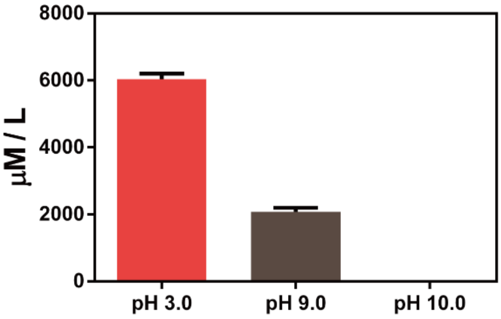

Figure S4. HPLC quantification of S_8 produced from decomposition of thiosulfate. Thiosulfate produces more S_8 at pH 3.0 and does not decompose at pH 10.0.
